# Laser-CMT Hybrid Welding-Brazing of Al/Steel Butt Joint: Weld Formation, Intermetallic Compounds, and Mechanical Properties

**DOI:** 10.3390/ma12223651

**Published:** 2019-11-06

**Authors:** Yuxin Chen, Zhibin Yang, Chunyuan Shi, Zhibin Xin, Zitong Zeng

**Affiliations:** 1 School of Materials Science and Engineering, Dalian Jiaotong University, Dalian 116028, China; 2 Engineering Department of CRRC Sifang Co., Ltd., Qingdao 266111, China

**Keywords:** laser-CMT hybrid welding-brazing, aluminum alloy, stainless steel, intermetallic compound, mechanical property

## Abstract

6A01-T5 aluminum alloy and SUS301L-DLT austenitic stainless steel sheets were welded by a laser-cold metal transfer (CMT) hybrid welding-brazing method with ER5183 filler wire. We researched the weld forming, intermetallic compounds, and mechanical character, which are influenced by laser power, wire feeding speed, and welding speed. Well-formed joints with uniformly distributed interface layers were obtained under certain parameters. The spreading and wetting distance on the steel upper surface increased initially and then decreased as the laser power increased, and increased progressively as the wire feeding speed increased or welding speed decreased. There were both Fe_2_Al_5_ and Fe_4_Al_13_ in the interfacial intermetallic compounds (IMCs) layer. The thickness was controlled to within 2.0–6.9 µm. The thickness of the IMCs layer increased as the heat input increased; however, the increasing rate decreased gradually. The tensile strength of the joints was not only completely dependent on the thickness of the IMCs, but also on the spreading and wetting distance on the steel surface. The highest tensile strength could reach up to 188.7 MPa, which is about 77.1% of that of the base aluminum alloy. The tensile sample fracture occurred at the IMCs layer, and regional metallurgical bonding happened in the interface layer.

## 1. Introduction

The composite structure of aluminum alloy and stainless steel has been expected to be applied in many modern manufacturing industries because it can simultaneously take advantage of the light weight of the aluminum alloy and high steel strength. [1,2]. On account of the large differences in physical properties between aluminum alloy and stainless steel, it is very difficult to obtain a high-quality Al/steel joint using the traditional fusion welding method [3,4]. In addition, some brittle and hard Fe-Al intermetallic compounds (IMCs) form while welding that decrease the mechanical properties of the joints [5,6].

Welding-brazing has been considered as an effective way to connect aluminum alloy to stainless steel, as well as other dissimilar metals [7]. During the welding-brazing process, the aluminum alloy and stainless steel are in liquid and solid state, respectively, and the growth of the IMCs is suppressed effectively [8,9,10,11,12]. At present, several welding-brazing methods have been proposed to join the dissimilar Al/steel metals, such as: tungsten inert gas (TIG) welding, metal inert gas (MIG) welding-brazing, laser beam welding-brazing, cold metal transfer (CMT) welding-brazing, laser-arc hybrid welding-brazing, and so on [13,14].

CMT welding is a new type of MIG welding method with ultra-low heat input, and its arc length is particularly stable during the welding process because the system will adjust the arc length automatically according to the situation [15,16,17]. Madhavan et al. [18] researched the microstructures and tensile strength of the Al/steel CMT welding-brazing joints. The results indicated that the phases of Fe_2_Al_5_ and FeAl_3_ were found in the interface and the thickness of the IMCs layer had a greater impact on tensile property. Compared to traditional MIG welding-brazing, CMT welding is more suitable for joining dissimilar Al/steel metals because the thickness of the IMCs layer is thinner and more uniform than that of MIG welding-brazing [19,20]. Compared with the MIG welding-brazing method, laser welding-brazing of Al/steel joints had a faster heating and cooling speed and therefore greatly decreased the growth tendency of the IMCs, but the interface reaction was still inhomogeneous [9,21]. Meanwhile, the defects of undercut and poor spreading and wetting were the major problems faced by the laser melt-brazed joints [22].

Laser-arc hybrid welding-brazing has all the advantages of both laser and arc welding-brazing, and is currently a major research hotspot [23,24,25]. Lei et al. [26] realized high-efficiency welding-brazing of dissimilar Al/steel metals by laser-MIG hybrid welding-brazing, where the thickness of the IMCs layer was controlled within 1.5–4 µm. The CMT welding method provides the possibility to improve the quality of the Al/steel weld-brazed joints [27]. However, at present, very few studies have reported the welding of dissimilar Al/steel metals using a laser-CMT hybrid welding-brazing method.

In this study, to research the laser-CMT hybrid welding-brazing characteristics of dissimilar Al/steel metals, the effects of laser power, wire feeding speed, and welding speed on the weld formation, intermetallic compounds, and mechanical properties of the weld-brazed joints were investigated in detail.

## 2. Materials and Experimental Details

**Figure 1:**
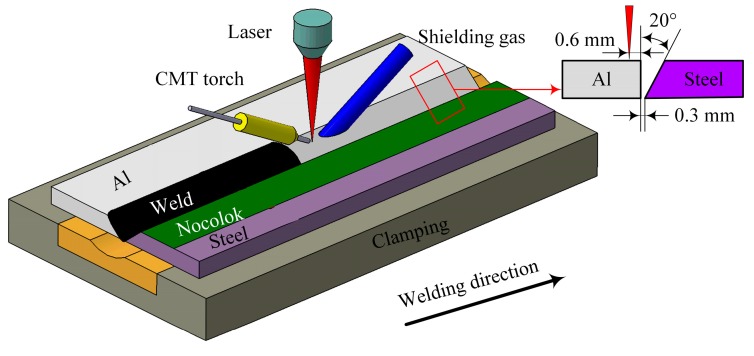
Schematic diagram for experimental set-up and groove geometry of the specimens. CMT: cold metal transfer.

6A01-T5 aluminum alloy (Yankuang Group, Zoucheng, China) and SUS301L-DLT stainless steel (Yankuang, Zoucheng, China) were selected as parent metals, whose dimensions were 100 × 50 × 2.5 mm^3^ and 100 × 50 × 2 mm^3^, respectively. A V-groove with an angle of 20 degrees was chamfered on the stainless steel edge, as shown in Figure 1. ER5183 wire with a diameter of 1.2 mm was used as filler wire. Table 1 and Table 2 show the chemical composition of the base metals and filler wire.

Before laser-CMT hybrid welding-brazing, the stainless steel was cleaned with acetone; in the meantime, the aluminum alloy was cleaned with NaOH and HNO_3_. After the treatment, the welding test was carried out within 24 h. A fine paste made of Nocolok Al-flux (Guangzhou, China) and acetone was coated on the V-groove surface of the stainless steel; the thickness of the coating was about 20–30 µm. The parent metals were fixed on the workbench, and the pitch was kept at 0.3 mm (as shown in Figure 1).

We employed an IPG YLS-6000 fiber laser (Burbach, Germany) in combination with a FRONIUS TPS 500i CMT welding machine (Wels, Austria) to conduct the laser-CMT hybrid welding test. The fiber laser had an emission wavelength of 1.06 µm, the laser beam passed through a focusing lens with a focal length of 300 mm, and was finally focused into a spot with a diameter of 0.2 mm. The KUKA 30HA robot (Augsburg, Germany) combined the laser head and the wire feed tube so that they could run synchronously to achieve the paraxial laser-CMT hybrid mode. During the welding-brazing process, the angle between the laser and the workpiece was 80°, and the CMT torch was 60°. The focus location was +5 mm to the upper surface of the parent metal and the laser beam offset was set 0.6 mm on the aluminum alloy (as shown in Figure 1); the distance between the laser focal point and the filler wire was 3 mm. The shielding gas was argon with purity of 99.999%, and its flow rate was 20 L/min. The welding parameters adopted in the experiments were as follows: laser power, 1.9–2.2 kW; welding speed, 0.8–1.4 m/min; wire feeding speed, 4–5.5 m/min; and the CMT current and CMT voltage were adjusted automatically according to the wire feeding speed.

After welding-brazing, standard samples (20 × 10 mm^2^) were cut from test pieces along the weld cross section, polished, and corroded with Keller’s reagent (1 mL HF + 1.5 mL HCl + 2.5 mL HNO_3_ + 95 mL H_2_O) for about 10 s at room temperature. The macroscopic appearance was observed by a 3D video microscope (KEYENCE VHX-1000, Osaka, Japan), and the spreading distance and wetting angle of the molten metals on the steel surface were regarded as evaluation criteria of spreadability and wettability on the steel upper surface. The tensile specimens were prepared according to the ISO 4136: 2001standard. The tensile test was carried out using a universal stretching machine (WDW-300E, Jinan, China) at a rate of 1 mm/min at room temperature, and the tensile strength was the average of three specimens. The interfacial microstructures and fracture morphologies were characterized using a scanning electron microscope (SEM) (ZEISS, SUPRA 55, Jena, Germany).

## 3. Results and Discussion

### 3.1. Effects of Welding Parameters on Weld Shape

**Figure 2:**
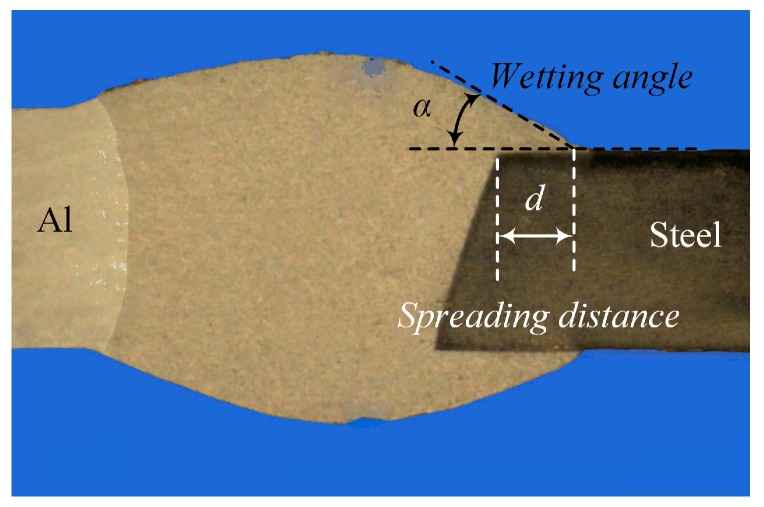
Definitions of spreading distance and wetting angle.

The weld shape is usually the first critical aspect to be considered to improve the joint quality. Defects such as undercut, poor spreading, and wetting significantly affect the mechanical properties of the weld-brazed joints. Therefore, the effects of the main welding parameters on the weld-brazed joints were studied, including wire feeding speed, laser power, and welding speed. The focus of attention in this work is on the effects of the above parameters on spreading distance (*d*) and wetting angle (*α*) on the stainless steel surface; their definitions are given in Figure 2.

#### 3.1.1. Wire Feeding Speed

**Figure 3:**
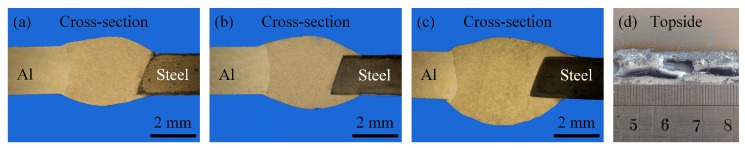
Effects of wire feeding speed on the weld shape: (**a**) 4.0 m/min; (**b**) 4.5 m/min; (**c**) 5.0 m/min; (**d**) 5.5 m/min.

**Figure 4:**
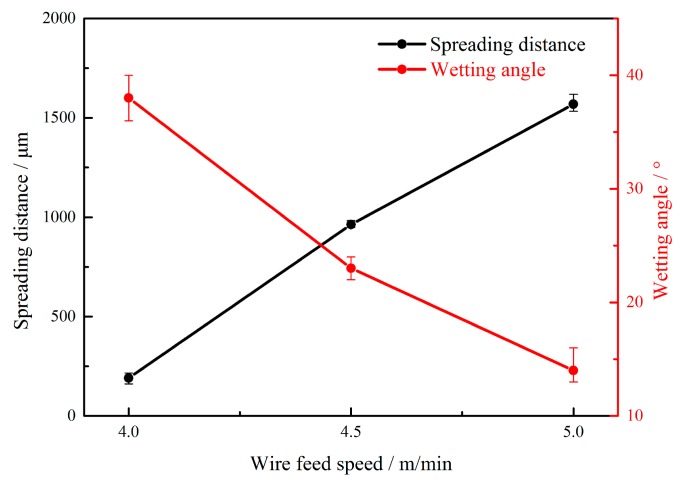
Spreading distance and wetting angle with different wire feeding speeds.

During the laser-CMT hybrid welding-brazing process, the wire feeding speed not only determines the values of the heat input, but also directly determines the spreading and wetting status of the weld. The effects of the wire feeding speed on the weld shape were as shown in Figure 3. The wire feeding speeds were set as 4.0, 4.5, and 5.0 m/min, and the corresponding laser power and welding speed were adjusted to 2.0 kW and 1.2 m/min, respectively. The well-formed weld-brazed joints were all obtained under the above three different wire feeding speeds, without undercut, porosity, or crack defects, as shown in Figure 3a–c. As the wire feeding speed increased, the spreading and wetting status on the stainless steel improved, and the weld looked like a plier clamping the stainless steel as shown in Figure 3c. The reason was that when welding, as the wire feed speed increased, the heat input would increase, and the spreadability and wettability of the molten metal were enhanced accordingly. The spreading distance was increased as wire feeding speed increased. In contrast, the wetting angle was decreased as wire feeding speed increased, as shown in Figure 4. Note that the problem of burn through occurred when the wire feeding speed was too high, as shown in Figure 3d.

#### 3.1.2. Laser Power

**Figure 5:**
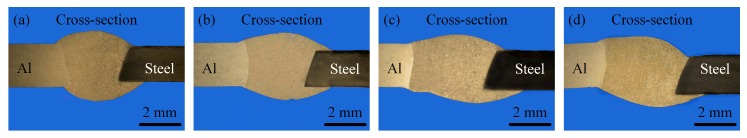
Effects of laser power on the weld shape:; (**a**) 1.9 kW; (**b**) 2.0kW; (**c**) 2.1 kW; (**d**) 2.2 kW.

**Figure 6:**
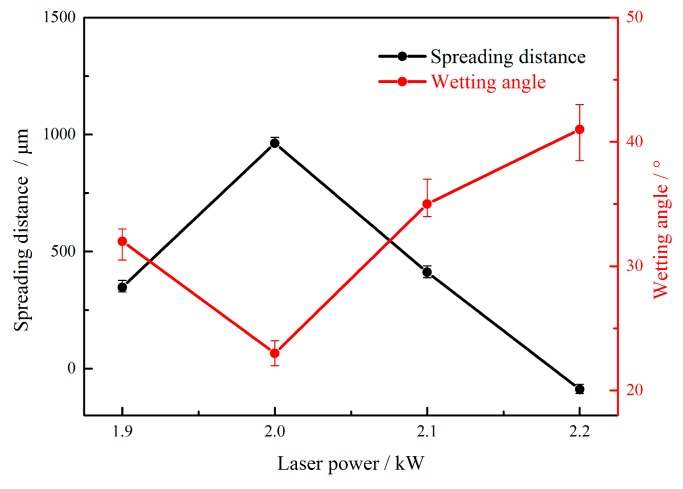
Spreading distance and wetting angle with different laser powers.

Figure 5 shows the effects of laser power on the weld shape. The laser powers were set as 1.9 kW, 2.0 kW, 2.1 kW, and 2.2 kW, and the corresponding wire feeding speed and welding speed were adjusted to 4.5 m/min and 1.2 m/min, respectively. No porosities and cracks appeared in the weld-brazed joints; however, a slight undercut was observed when the laser power was too large (Figure 5d). The reason was that, during the laser-CMT hybrid welding-brazing process, the laser beam set an offset of 0.6 mm on the aluminum alloy (Figure 1) and had a preheating function on the stainless steel. Therefore, it could promote the spreading and wetting. However, increasing weld width resulted in the reinforcement of the weld topside decreasing and the wetting status becoming poor. The effects of laser power on the weld spreading distance and wetting angle are shown in Figure 6. The spreading distance increased and wetting angle decreased as laser power increased when the laser power was less than 2.0 kW, and vice versa when the laser power was greater than 2.0 kW.

#### 3.1.3. Welding Speed

**Figure 7:**
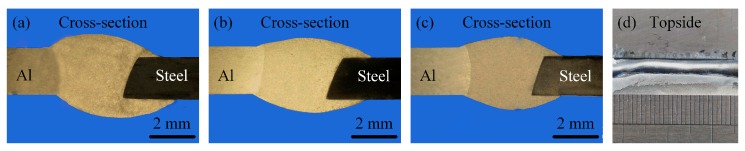
Effects of welding speed on the weld shape. (**a**) 0.8 m/min; (**b**) 1.0 m/min; (**c**) 1.2 m/min; (**d**) 1.4 m/min.

**Figure 8:**
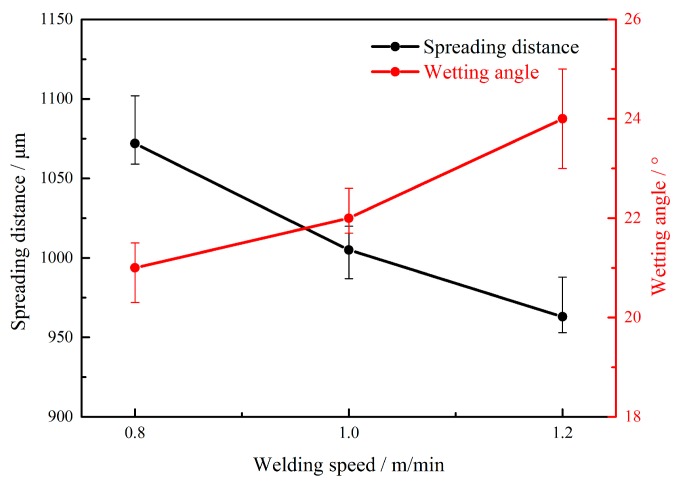
Spreading distance and wetting angle with different welding speeds.

Figure 7 and Figure 8 show the effects of welding speed on the weld shape, spreading distance, and wetting angle, respectively. The welding speed was set as 0.8, 1.0, 1.2, and 1.4 m/min, and the corresponding wire feeding speed and laser power were adjusted to 4.5 m/min and 2.0 kW, respectively. As shown in Figure 7 and Figure 8, the spreading and wetting decreased as the welding speed increased, though the defect of poor fusion appeared as shown in Figure 7d. The above changes were due to the fact that the heat input had a weaker preheating function on the upper surface of the stainless steel as the welding speed increased, and the melted aluminum alloy solidified before it could spread and wet on the stainless steel surface. Meanwhile, the filler content per unit weld length of the wire decreased as the welding speed increased, which was the other reason for this phenomenon.

### 3.2. Effects of Heat Input on IMCs Layer Thickness

The weakest link of the Al/steel welded joint is located at the interface of the IMCs layer. Therefore, the mechanical properties of the joints were mainly dependent on the species, morphologies, and thicknesses of the IMCs. The formation and growth of the IMCs were affected by the heat input during the welding process. In this work, the microstructure of the IMCs under different heat inputs was investigated by SEM. In order to facilitate the comparison results, the observed interface positions were adjusted to the longitudinal direction.

**Figure 9:**
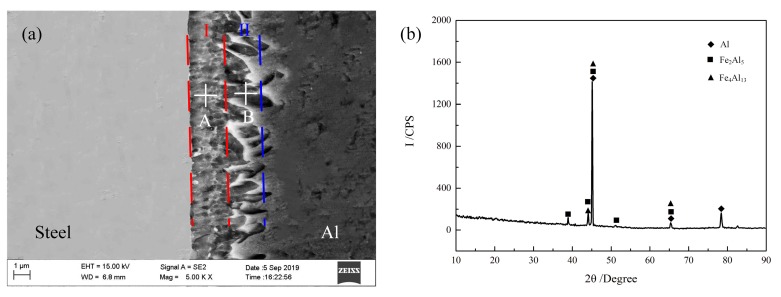
Microstructure and XRD results of the intermetallic compounds (IMCs) layer (laser power 2.1 kW, welding speed 1.2 m/min, wire feeding speed 4.5 m/min). (**a**) Microstructure; (**b**) XRD results.

The typical Al/steel interfacial microstructure is shown in Figure 9a. It was found that the IMCs could be roughly divided into two representative morphologies: one near the steel side had smooth morphology, and another near the seam exhibited a zigzag feature, which were respectively designated as I-layer and II-layer. To confirm the IMCs’ phase compositions, XRD analysis in the two representative layers was carried out in this work, as shown in Figure 9b. According to the recent research results of Van Alboom et al., Fe_4_Al_13_ is more suitable than FeAl_3_ [28]. Therefore, we can derive that there were both Fe_2_Al_5_ and Fe_4_Al_13_ in the interfacial intermetallic compounds layer—the first near the steel side and another near the seam.

**Figure 10:**
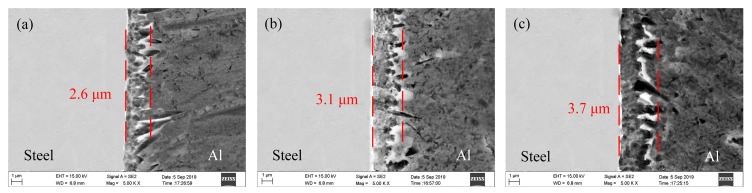
Microstructure of the IMCs layer with different wire feeding speed: (**a**) 4.0 m/min; (**b**) 4.5 m/min; (**c**) 5.0 m/min.

**Figure 11:**
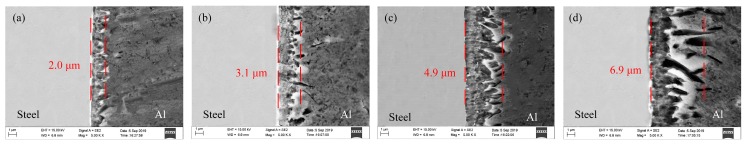
Microstructure of the IMCs layer with different laser power: (**a**) 1.9 kW; (**b**) 2.0 kW; (**c**) 2.1 kW; (**d**) 2.2 kW.

**Figure 12:**
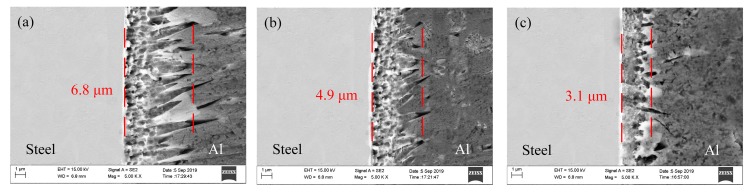
Microstructure of the IMCs layer with different welding speed: (**a**) 0.8 m/min; (**b**) 1.0 m/min; (**c**) 1.2 m/min.

**Figure 13:**
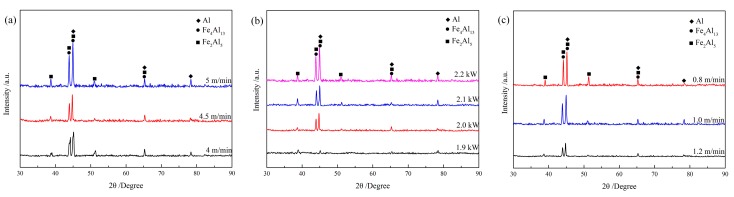
Phase composition of the IMCs layer under different welding parameters: (**a**) wire feeding speed; (**b**) Laser power; (**c**) Welding speed.

The effects of wire feeding speed, laser power, and welding speed on the thicknesses of the IMCs layer are given in Figure 10, Figure 11 and Figure 12, respectively. Through integrated comparison, it was found that the thickness of IMCs layers increased significantly with increasing wire feeding speed or laser power, or decreasing welding speed. In this work, the IMCs layer thickness ranged from 2.0 to 6.9 µm. The results indicate that the formation and growth of the IMCs layer were directly affected by the heat input, because as the heat input increased, the interface reacted more intensely and the longer times spent at high temperature resulted in longer growth time of the IMCs. The XRD results of the IMCs layer under different welding parameters are shown in Figure 13. They indicate that all of the IMCs layers consisted of Fe_2_Al_5_ and Fe_4_Al_13_, and their diffraction intensity increased gradually as the heat input increased. The enhanced diffraction intensity also manifested an increase in the thickness of the IMCs layer.

### 3.3. Mechanical Properties

**Figure 14:**
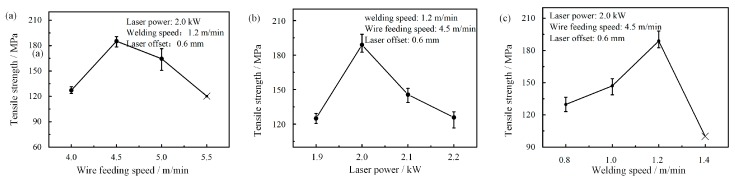
Effects of welding-brazing parameters on the tensile strength of the joints: (**a**) Wire feeding speed; (**b**) Laser power; (**c**) Welding speed.

**Figure 15:**
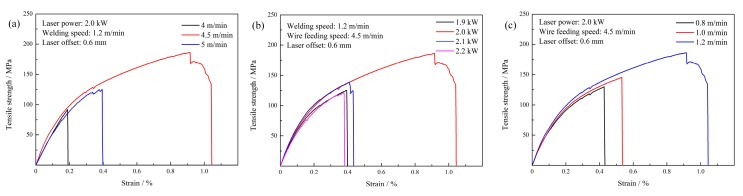
Stress-strain curves of the tensile test with different welding parameters: (**a**) Wire feeding speed; (**b**) Laser power; (**c**) Welding speed.

Figure 14 shows the effects of welding-brazing parameters on the tensile strength of the joints. It was found that the highest tensile strength (average about 188.7 MPa, which is 77.1% of the aluminum alloy base metal (245 MPa)) could be obtained when the wire feeding speed, laser power, and welding speed were 4.5 m/min, 2.0 kW, and 1.2 m/min, respectively. The stress-strain curve is shown in Figure 15. It indicates that the fracture features changed from brittle to ductile as the tensile strength increased. In general, the tensile strength decreased as heat input increased, which indicated that the thickness of IMCs was the main factor affecting the tensile strength. Thinner IMCs could be obtained when the feeding speed was 4.0 m/min or laser power was 1.9 kW. However, the tensile strength neither increased nor decreased. This is because the spreading and wetting worsened under the welding-brazing parameters, as shown in Figure 3a and Figure 5a. They indicated that the better spreading and wetting provided the additional adhesion strength for the laser-CMT weld-brazed joints. Therefore, it can be assumed that the tensile strength of the joints is not completely dependent on the thickness of the IMCs, and is also dependent on the spreading and wetting distance on the steel surface.

**Figure 16:**
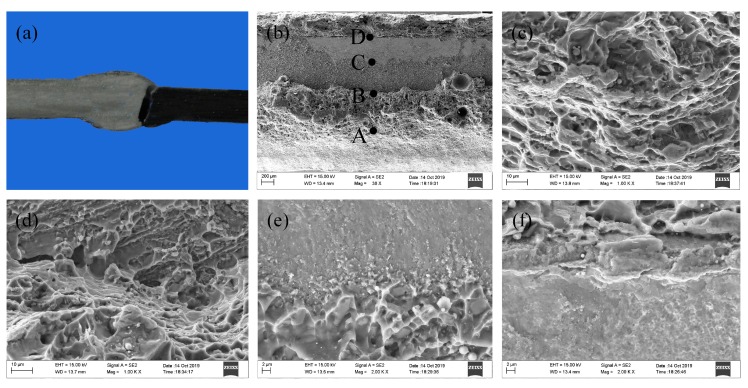
Macro-fracture location and micro-fracture morphologies of the tensile specimen: (**a**) Macro-fracture location; (**b**) Micro-fracture morphology; (**c**) Larger version of domain A; (**d**) Larger version of domain B; (**e**) Larger version of domain C; (**f**) Larger version of domain D.

The macro-fracture location and micro-fracture morphologies of the tensile specimen are shown in Figure 16. The fracture initiated at the weld toe during tensile testing, and the specimens were fractured near the interface as shown in Figure 16a. Due to the spreading and wetting of the melted filler wire, the topside and backside of the weld fracture morphologies exhibited typical ductile fracture, as shown in Figure 16b,c,f. From Figure 16e, no dimples could be observed on the fracture surface, and an obvious brittle fracture feature was seen. This indicated that the sample fractured along the IMCs layer, which was the weakest area of the weld-brazed joint. Near the domain C, some small and shallow dimples appeared as shown in Figure 16e, which indicated that metallurgical bonding occurred in the interface layer, which was beneficial in improving the joint tensile strength. 

## 4. Conclusions

(1) Well-formed Al/steel joints without the defects of undercut, porosities, and cracks could be obtained with suitable processing parameters by a laser-CMT welding-brazing method. The spreading and wetting distance on the steel surface increased initially and then decreased as the laser power increased, and increased progressively as the wire feeding speed increased or welding speed decreased.

(2) There were both Fe_2_Al_5_ and Fe_4_Al_13_ in the interfacial IMCs layer, the first near the steel side and another near the seam, and the thickness was controlled to within 2.0–6.9 µm. The thickness of the IMCs layer increased as the heat input increased, and the rate of rise decreased gradually.

(3) The highest tensile strength could reach up to 188.7 MPa, which was about 77.1% of the base aluminum alloy. The tensile strength of the joints was not completely dependent on the thickness of the IMCs; it was also dependent on the spreading and wetting distance on the steel surface. The tensile sample fractured along the IMCs layer, and regional metallurgical bonding occurred in the interface layer.

## Figures and Tables

**Table 1:** Chemical composition of 6A06-T5 aluminum alloy and ER5183 filler wire (wt.%).

Materials	Si	Fe	Cu	Mn	Mg	Cr	Zn	Ti	Al
6A01-T5	0.60	0.25	0.20	0.40	0.68	0.20	0.10	0.08	Bal.
ER5183	0.40	0.15	≤0.05	0.05	3.5	0.20	0.10	0.10	Bal.

**Table 2:** Chemical composition of SUS301L-DLT stainless steel (wt.%).

C	Si	Mn	P	S	Ni	Cr	N	Fe
<0.03	<1.00	<2.00	<0.045	<0.03	6–8	16–18	<0.2	Bal.
